# Pan-immune-inflammation and its dynamics: predictors of survival and immune-related adverse events in patients with advanced NSCLC receiving immunotherapy

**DOI:** 10.1186/s12885-023-11366-4

**Published:** 2023-10-06

**Authors:** Yiqun Chen, Lingyan Gong, Pengyang Gu, Yiwen Hua, Yingfang Sun, Songshi Ni, Xiaoyu Zhou, Zhiyuan Tang

**Affiliations:** 1 Department of Respiratory and Critical Care Medicine, Affiliated Hospital of Nantong University, Medical School of Nantong University, Nantong, 226001 China; 2 Department of Pharmacy, Affiliated Hospital of Nantong University, Pharmacy School of Nantong University, Nantong, 226001 China

**Keywords:** Non-small-cell lung cancer, Immune-checkpoint inhibitors, Pan-immune-inflammation value, Immune-related adverse events, Blood biomarkers, Dynamics

## Abstract

**Objectives:**

Pan-immune-inflammation value (PIV) is defined by the neutrophil, platelet, monocyte, and lymphocyte counts and is associated with immune-checkpoint inhibitor (ICI) therapy outcomes in advanced non-small cell lung cancer (aNSCLC). However, PIV is dynamic under therapy and its longitudinal assessment may help predict efficacy. This study investigated the impact of baseline PIV and its dynamics on ICI efficacy and its immune-related adverse events (irAEs). The study additionally attempted to understand the biological significance of PIV.

**Patients and methods:**

This retrospective study analyzed the clinical data of 269 consecutive patients with aNSCLC. PIV was calculated at baseline and at weeks 3–4 to determine its association with overall survival (OS), progression-free survival (PFS), and irAEs.

**Results:**

Results revealed that low baseline PIV was positively correlated with the incidence of irAEs. Moreover, a low PIV at baseline was significantly associated with a prolonged PFS (median PFS: 10 vs. 7 months, *p =* 0.0005) and OS (median OS: 29 vs. 21 months, *p <* 0.0001). When the PIV at baseline and weeks 3–4 was considered together, its low dynamics correlated with a higher incidence of irAEs (*p =* 0.001), a longer PFS (median PFS, 9 vs. 6 months, *p =* 0.012), and a longer OS (median OS; 28 vs. 21 months, *p =* 0.002).

**Conclusion:**

Thus, PIV at baseline and its dynamics are novel and potent predictors of irAEs, PFS, and OS in patients with aNSCLC receiving immunotherapy. Moreover, the PIV dynamics may be an effective, novel surrogate marker to dynamically observe the efficacy of immunotherapy.

## Introduction

Lung cancer is the second most commonly occurring tumor and is the leading cause of cancer-related mortality worldwide [1]. Particularly, non-small cell lung cancer (NSCLC) accounts for 85% of all lung cancers [2, 3]. Compared with surgery, radiotherapy, or chemotherapy, immunotherapy has become a hot topic for treating NSCLC owing to the considerable, recent advancements in this therapy. Moreover, it has been shown that patients with advanced NSCLC (aNSCLC) treated with immunotherapy have longer progression-free survival (PFS) and overall survival (OS) than those treated with conventional chemotherapy [4, 5].

Despite its advantages, immunotherapy still is a double-edged sword. Unlike traditional anti-tumor drugs, immunotherapy does not directly kill tumor cells; instead, it regulates the body’s immune function [6]. By changing the inherent relationship between immune and tumor cells, immunotherapy alters the tumor microenvironment (TME) such that immune cells kill cancer cells [7, 8]. However, immunotherapy is associated with a unique set of adverse events, called immune-related adverse events (irAEs) [9–11]. These result from treatment-induced over-activation of the immune system, which causes injury to normal tissues, resulting in more serious treatment complications and thus limiting the clinical use of immune-checkpoint inhibitors (ICIs). To add to this dilemma, there is a lack of biomarkers to predict the occurrence and severity of irAEs. Studies have shown that in the process of immunotherapy for non-small cell lung cancer, there is a correlation between the occurrence of irAEs and good survival [12]. Therefore, if alternative biomarkers for predicting irAEs can be found and the advantages and disadvantages of immunotherapy can be weighed before treatment, patients will benefit from it, reducing the occurrence of immune adverse events and get the possibility of personalized immunotherapy. Thus, more studies are required to identify factors associated with irAEs.

It is well known that inflammation impacts every step of tumorigenesis, from initiation and tumor promotion to metastatic progression. Reportedly, liquid biopsy is a promising tool for identifying predictive biomarkers for immunotherapy [13]. According to previous studies, peripheral blood parameters may efficiently predict responses to ICIs in multiple malignancies. Particularly, inflammation-related markers, such as the systemic inflammation immune index (SII) [14], neutrophil-to-lymphocyte ratio (NLR) [15], platelet-to-lymphocyte ratio (PLR), monocyte-to-lymphocyte ratio (MLR) [16], and derived NLR (dNLR) [17] can all be used as potential markers of tumor response to ICIs.

Recently, the dynamics of biomarkers have been studied to some extent for determining the prognosis of cancer treatments [18–21]. In the context of immunotherapy for NSCLC, several factors have been reported for monitoring efficacy and predicting clinical prognosis; these factors include multiple mechanisms, including dynamic immune TME profiles [22], PD-L1 expression [23], radiomics [24], tumor mutation burden, and immunoinflammatory indicators [25, 26]. Therefore, unlike in the past, when pathological biopsy of primary tumor or blood tests were performed before treatment, studying the dynamics of peripheral blood biomarkers is worthy of recognition and further investigation in the prognostic assessment of immunotherapy for NSCLC.

More recently, a new comprehensive marker called the pan-immune-inflammatory value (PIV) [27], which incorporates neutrophil, platelet, monocyte, and lymphocyte counts, showed a strong association with PFS and OS. Moreover, PIV was found to outperform other well-established immune biomarkers, such as NLR and PLR, in predicting patient outcomes [28]. In addition, a study has validated the role of PIV dynamics for disease monitoring in patients with metastatic colorectal cancer [29]. Therefore, the present study aimed to assess the predictive value of PIV and its dynamics in patients with aNSCLC.

## Patients and methods

### Patients

The study retrospectively enrolled patients with aNSCLC who were consecutively treated at the Affiliated Hospital of Nantong University, China, between January 2019 and December 2022. All patients had undergone immunotherapy with nivolumab 3 mg/kg every 2 weeks or pembrolizumab 200 mg every 3 weeks until unacceptable toxicity, or death from any cause. Patients who only received other antitumor therapies, such as chemotherapy, targeted therapy, or radiotherapy, were excluded from the study. Only patients with a minimum follow-up of 12 months (until December 2022) were enrolled, owing to the high probability of developing irAEs within the 1– 6 months of immunotherapy [30] and the need for sufficient time for survival assessment.

### Data collection

Data on the following patients’ characteristics were extracted: age, sex, smoking history, histology, cancer stage, and the number of metastatic sites. In addition, laboratory data such as complete blood cell counts (e.g., neutrophils, lymphocytes, monocytes, and platelets) at baseline (within 7 days before the administration of immunotherapy, defined as T1) and at the endpoint (3–4 weeks after the first dose, defined as T2) were extracted from the electronic medical records. PIV was calculated as (neutrophil count × platelet count × monocyte count)/lymphocyte count. SII was calculated as (neutrophil count × platelet count)/lymphocyte count. NLR was calculated as neutrophil count /lymphocyte, and PLR was calculated as platelet count/lymphocyte count. MLR was calculated as monocyte count/lymphocyte count and d-NLR as neutrophil count/(leucocytes count – neutrophil count). The cutoff values for PIV, SII, NLR, PLR, MLR, and d-NLR at baseline as dichotomous variables were intercepted according to the receiver-operating characteristic curve (PIV < 288.1; SII < 784.1; NLR < 3.163; PLR < 0.420; dNLR < 2.316). The dynamic change in PIV was calculated by subtracting PIV (T1) from the absolute PIV (T2). The cutoff values for the dynamic changes in PIV as dichotomous variables were determined using the X-title software (PIV dynamics < 608.2). PFS was determined as the duration from the date of treatment initiation to the date of disease progression or patient death from any cause. OS was defined as the duration from the date of treatment initiation to the date of death from any cause. Tumor responses were assessed using the Response Evaluation Criteria in Solid Tumors guidelines (version 1.1) every 10 ± 2 weeks. This study was approved by the board/ethics committee of the Affiliated Hospital of Nantong University, and exception to the requirement of informed consent was approved (Ethic Number: 2018-K020).

### Statistical analysis

Variability was compared using Fisher’s exact test for categorical variables and Mann-Whitney or Wilcoxon signed-rank tests for continuous variables. Box charts and line charts were used to illustrate the differences and trends between the two important time points of PIV. The association between blood biomarkers and the onset of irAEs was analyzed by logistic regression models. For data with binary data as the outcome variable, the cut-off value of the continuous independent variable is generally determined by ROC analysis. For dichotomous outcome variables with temporal dimensions, we use X-title to determine the cut-off value [31]. Cox regression models were used to determine the risk factors for PFS and OS. Moreover, PFS and OS were estimated using the Kaplan-Meier method and were compared using the log-rank test. Statistical significance was defined as a two-sided p-value of < 0.05. All statistical analyses were performed using X-tile versions 3.6.1, SPSS 20.0 (SPSS Inc., Chicago, IL) and GraphPad Prism version 9.2.0 (GraphPad Inc., San Diego, CA).

## Results

### Clinical characteristics

The clinical characteristics of 269 patients are outlined in Table 1. The median age of patients was 67 (41–87) years, with 232 (86.2%) patients being male. The most common histological type of NSCLC was adenocarcinoma (n = 164; 61.0%), followed by squamous carcinoma (n = 104; 38.6%) and a rare type of sarcoma (n = 1; 0.4%). Of all patients, 18 (6.7%) had an Eastern Cooperative Oncology Group performance status of 2. Overall, 215 (55.2%) patients had undergone testing for tumor PD-L1 expression; of these, 147 (68.4%) and 68 (31.6%) patients tested positive and negative for PD-L1, respectively. The median number of times patients underwent treatment at baseline was five, with 168 (62.5%) patients being treated with pembrolizumab and 101 (37.5%) with nivolumab. For blood biomarkers, the median PIV was 387 (17.8–9420), NLR was 3.37 (0.844–79.7), PLR was 164 (36.4–1490), SII was 682 (142.0–17300.0), PLR was 164 (36.4–1490), and dNLR was 2.07 (-1.34–31.4).

**Table 1:** Clinical characteristics

	Overall (N = 269)
**Gender**	
Female	37.0 (13.8%)
Male	232.0 (86.2%)
**Age**	
Median (range)	67.0 [41.0, 87.0]
**Smoker**	
No	83.0 (30.9%)
Yes	186.0 (69.1%)
**Histology**	
Squamous carcinoma	164.0 (61.0%)
Adenocarcinoma	104.0 (38.6%)
Sarcoma	1.0 (0.4%)
**Stage**	
III	92.0 (34.2%)
IVA	118.0 (43.9%)
IVB	59.0 (21.9%)
**Number of treatment with ICIs**	
Median (range)	5.00 [2.00, 50.0]
**PD-1/PD-L1 TPS**	
< 1%	68.0 (25.3%)
> 1%	147.0 (54.6%)
Unknown	54.0 (20.1%)
**Number of metastatic sites**	
<3	210.0 (78.1%)
≥ 3	59.0 (21.9%)
**ICIs agent**	
Pembrolizumab	168.0 (62. 5%)
Nivolumab	101.0 (37.5%)
**ECOG.PS**	
0	115.0 (42.8%)
1	136.0 (50.6%)
2	18.0 (6.7%)
**PIV**	
Median (range)	387.0 [17.8, 9420]
**SII**	
Median (range)	682.0 [142, 17,300]
**NLR**	
Median (range)	3.37 [0.844, 79.7]
**PLR**	
Median (range)	164.0 [36.4, 1490]
**MLR**	
Median (range)	0.408 [0.04, 7.12]
**dNLR**	
Median (range)	2.07 [-1.34, 31.4]

irAEs, immune-related adverse events; ECOG/PS, Eastern Cooperative Oncology Group Performance Status; PD-1/PD-L1 TPS, Programmed Cell Death-1/Programmed Cell Death-Ligand 1 Tumor Proportion Score; ICIs, Immune Checkpoint Inhibitors; PIV, pan-immune-inflammatory value; SII, systemic immuneinflammation index; NLR, neutrophil to lymphocyte ratio; PLR, platelet to lymphocyte ratio; MLR, monocyte to lymphocyte ratio; dNLR, neutrophil to (leucocytes-neutrophils) ratio

### Summary of irAEs

In total, 89 (33%) patients reported irAEs (Table 2), with the most common being hyperthyroidism/hypothyroidism (19.7%), skin-related events (17.4%), liver injury (15.1%), and enteritis/diarrhea (15.1%). Among organ-related toxicities, dermatological and endocrine toxicities were the most commonly reported irAEs, followed by hepatologic and digestive disorders. A total of 89 events of all-grade irAEs were documented, of which 12 were grade ≥ 3 in severity. The most common grade ≥ 3 irAEs were rash and adrenal insufficiency. The grade ≥ 4 irAEs included myocarditis and complex infections because of hypoimmunity. However, very few grade ≥ 4 events were observed; but these may be resulted in death. The majority of patients with grade ≥ 3 irAEs were treated with steroids, and for a few patients with higher grade irAEs, immunotherapy was discontinued permanently. All patients who received immunosuppressive therapies achieved alleviation of their irAEs.

**Table 2:** Immune-related adverse events (n = 86)

			Grading		
irAE subtypes	Total	1	2	3	≥ 4
**Skin-related events**	15	5(34%)	7(47%)	2(13%)	1(6%)
**Pneumonitis**	7	2(28%)	4(57%)		1(15%)
**Enteritis/Diarrhea**	13	3(23%)	9(69%)	1(8%)	
**Endocrine**					
Hyperthyroidism/Hypothyroidism	17	7(41%)	10(59%)		
Adrenal insufficiency	3		1(33%)	2(67%)	
**Hepatology**	13	2(15%)	10(77%)	1(8%)	
**Nephrology**	4		4(100%)		
**Hematologic**					
Neutropenia	6	2(33%)	3(50%)	1(17%)	
Thrombocytopenia	5	2(40%)	3(60%)		
**Others**					
Myocarditis	1				1(100%)
Complex infections	1				1(100%)
Neuritis	1			1(100%)	

### Univariate and multivariate analyses of risk factors for irAEs

Findings regarding the association of irAE onset with baseline clinical features and with blood parameters are listed in Table 3. None of the baseline clinical features affected the overall risk of irAEs. For example, an increased number of treatment cycles was not significantly associated with a higher probability of developing irAEs (odds ratio [OR]: 0.969; 95% confidence interval [CI]: 0.917–1.024; *p =* 0.262). Moreover, no association was noted between PD-L1 expression and irAE onset (OR: 0.787; 95% CI: 0.406–1.524; *p =* 0.477). However, the following blood markers showed a significant correlation with the occurrence of irAEs: L-PIV (OR: 0.112; 95% CI: 0.062–0.202; *p <* 0.001), L-SII (OR: 0.129; 95% CI: 0.067–0.250; *p <* 0.001), L-NLR (OR, 0.294; 95% CI, 0.171–0.504; *p <* 0.001), L-PLR (OR: 0.164; 95% CI: 0.092–0.293; *p <* 0.001), L-MLR (OR: 0.229; 95% CI: 0.129–0.407; *p <* 0.001), and L-dNLR (OR: 0.299; 95% CI: 0.165–0.541; *p <* 0.001). Finally, multivariate analysis confirmed that L-PIV (OR: 0.235; 95% CI: 0.117–0.472; *p* < 0.001; Table 3), L-SII (OR: 0.393; 95% CI: 0.175–0.883; *p =* 0.024), and L-PLR (OR: 0.483; 95% CI: 1.055–4.500; *p =* 0.035) were the independent predictors of irAEs.

**Table 3:** Univariate and multivariate analyses of irAEs

Variable	OR	Univariate 95% CI	*p* Value	VIF	OR	Multivariate 95%CI	*p* Value
Gender	1.353	0.658–2.780	0.411				
Age	1.004	0.974–1.035	0.787				
Smoker	1.656	0.962–2.850	0.068				
Histology	0.787	0.453–1.366	0.394				
Stage	1.102	0.545–2.231	0.787				
Number of treatment with ICIs	0.969	0.917–1.024	0.262				
PD-1 TPS	0.787	0.406–1.524	0.477				
Number of metastatic sites	1.091	0.621–1.785	0.785				
ICIs agent	1.053	0.584–2.038	0.848				
ECOG/PS	1.048	0.686–1.602	0.827				
PIV(H/L)	0.112	0.062–0.202	**< 0.001**	1.976	0.235	0.117–0.472	**< 0.001**
SII(H/L)	0.129	0.067–0.250	**< 0.001**	2.511	0.393	0.175–0.883	**0.024**
NLR(H/L)	0.294	0.171–0.504	**< 0.001**	2.777			0.356
PLR(H/L)	0.164	0.092–0.293	**< 0.001**	1.571	0.483	0.240–0.972	**0.041**
MLR(H/L)	0.229	0.129–0.407	**< 0.001**	1.691			0.657
dNLR(H/L)	0.299	0.165–0.541	**< 0.001**	2.391			0.509

irAEs, immune-related adverse events; ECOG/PS, Eastern Cooperative Oncology Group Performance Status; PD-1/PD-L1 TPS, Programmed Cell Death-1/Programmed Cell Death-Ligand 1 Tumor Proportion Score; ICIs, Immune Checkpoint Inhibitors; PIV, pan-immune-inflammatory value; SII, systemic immuneinflammation index; NLR, neutrophil to lymphocyte ratio; PLR, platelet to lymphocyte ratio; MLR, monocyte to lymphocyte ratio; dNLR, neutrophil to (leucocytes-neutrophils) ratio; H/L, High/Low. ***P*** values were indicated in bold when statistical results were significant

In addition, determining collinearity using the variance inflation factor (PIV: 1.976; SII: 2.511; NLR: 2.777; PLR: 1.571; MLR: 1.691; dNLR: 2.391) revealed the absence of multicollinearity for blood biomarkers (Table 3).

### Impact of peripheral blood parameters on PFS and OS

Specific variables identified as by univariate analysis included the number of treatments with ICIs, PIV, SII, NLR, PLR, MLR, and dNLR (*p <* 0.05; Table 4). These variables were further analyzed using the multivariate model. Finally, only the number of treatments with ICIs (HR = 0.950, 95% Cl: 0.920–0.982, *p =* 0.002) and higher PIV (HR = 1.707, 95% Cl: 1.275–2.286, *p <* 0.001) were the independent prognostic factors for poor median PFS (Table 4).

**Table 4:** Univariate and Multivariable Cox proportional hazards model for PFS and OS

			PFS						OS			
Variable	HR	Univariate 95% CI	*p* Value	HR	Multivariate 95%CI	*p* Value	HR	Univariate 95% CI	*p* Value	HR	Multivariate 95%CI	*p* Value
Gender	1.431	0.974–2.103	0.068				0.661	0.305–1.433	0.294			
Age	0.999	0.983–1.016	0.922				1.019	0.994–1.045	0.144			
Smoker	0.849	0.624–1.156	0.298				0.943	0.588–1.511	0.808			
Histology	3,319	0.460-23.962	0.234				1.221	0.796–1.872	0.361			
Stage	0.882	0.613–1.271	0.501				0.546	0.309–0.962	**0.036**			0.797
Number of treatment with ICIs	0.958	0.928–0.990	**0.005**	0.950	0.920–0.982	**0.002**	0.982	0.949–1.016	0.289			
PD-1 TPS	1.459	0.990–2.151	0.057				1.663	0.930–2.973	0.086			
Number of metastatic sites	0.915	0.654–1.280	0.603				0.598	0.373–0.959	**0.033**	0.602	0.375–0.966	**0.035**
ICIs agent	0.767	0.572–1.030	0.078				0.868	0.561–1.344	0.526			
ECOG/PS	0.969	0.774–1.213	0.784				1.273	0.938–1.727	0.121			
PIV(H/L)	1.622	1.212–2.170	**0.001**	1.707	1.275–2.286	**< 0.001**	2.414	1.509–3.862	**< 0.001**	2.406	1.504–3.850	**< 0.001**
SII(H/L)	1.421	1.072–1.882	**0.014**			0.455	1.785	1.171–2.720	**0.007**			0.493
NLR(H/L)	1.361	1.025–1.808	**0.033**			0.446	1.555	1.008–2.398	**0.046**			0.614
PLR(H/L)	1.557	1.118–2.168	**0.009**			0.189	2.171	1.225–3.848	**0.008**			0.190
MLR(H/L)	1.424	1.076–1.886	**0.013**			0.226	2.124	1.386–3.255	**0.001**			0.055
dNLR(H/L)	1.389	1.046–1.844	**0.023**			0.185	1.817	1.193–2.769	**0.005**			0.057

PFS, Progression Free Survival; OS,Overall Survival; ECOG/PS, Eastern Cooperative Oncology Group performance status; PD-1/PD-L1 TPS, programmed cell death-1/ programmed cell death-ligand 1 Tumor Proportion Score; ICI,Immune checkpoint inhibitor; PIV, Pan-Immune-Inflammatory Value; SII, systemic immuneinflammation index; NLR, neutrophil to lymphocyte ratio; PLR, platelet to lymphocyte ratio; MLR, monocyte to lymphocyte ratio; dNLR, neutrophil to (leucocytes-neutrophils) ratio; H/L, High/Low. ***P*** values were indicated in bold when statistical results were significant

**Fig. 1:**
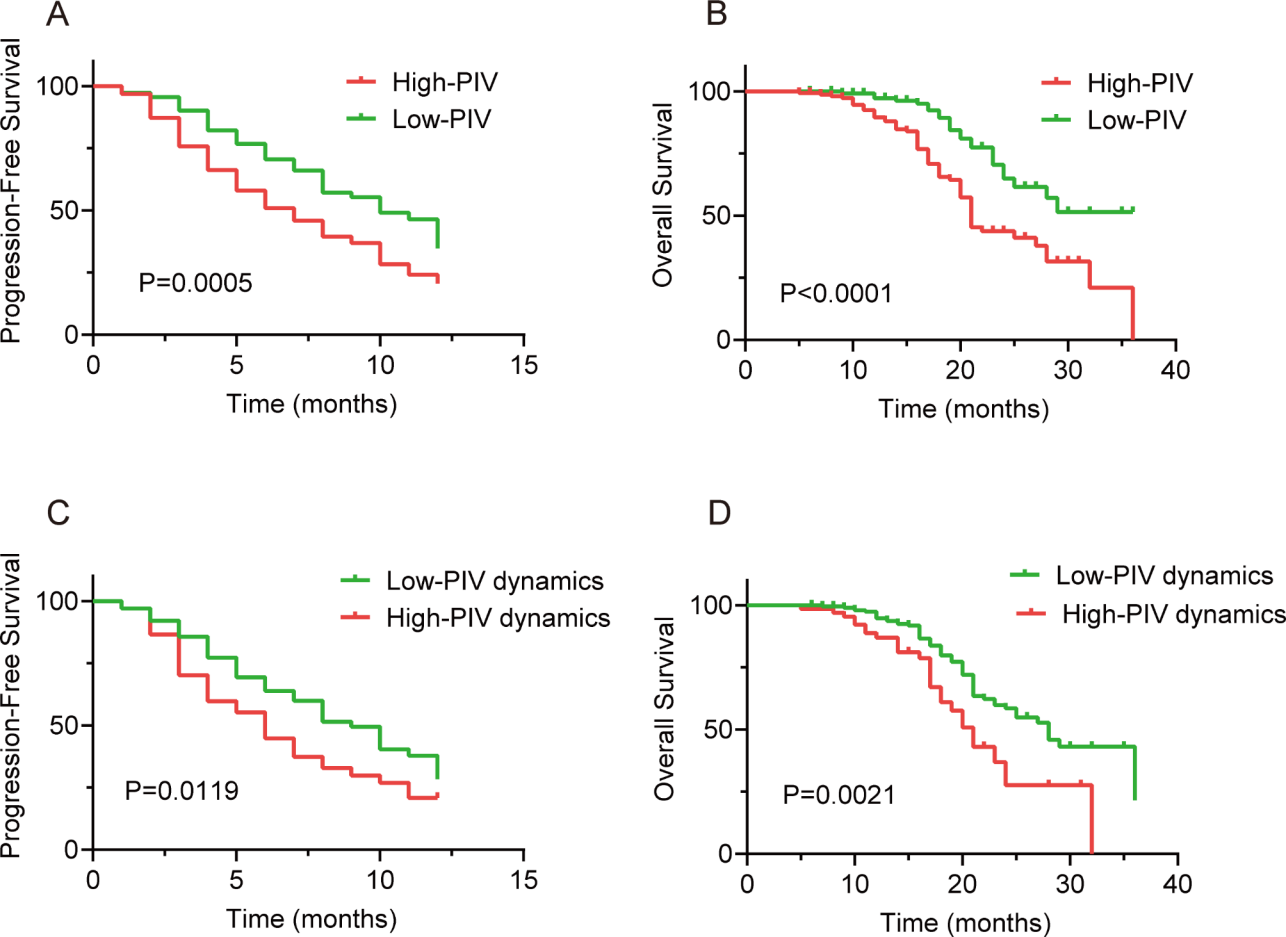
Kaplan-Meier survival estimates according to baseline PIV and PIV dynamics. (**A**) Progression-free survival according to PIV at baseline. (**B**) Overall survival according to PIV at baseline. (**C**) Progression-free survival according to PIV dynamics. (**D**) Overall survival according to PIV dynamics

A total of 196 tumor progression events were observed in 1 year of follow-up, with the median PFS being 7 months. The tumor progression events rate was 72.9%, with the median PFS being significantly longer in patients with low PIV (10 months, 95% CI: 8.6–11.4; Fig. 1A).

Univariate analysis demonstrated that stage, number of metastatic sites, PIV, SII, NLR, PLR, MLR, and dNLR were associated with OS (*p <* 0.05, Table 4). However, in multivariate analysis, only low PIV (HR = 2.414, 95% Cl: 1.509–3.862, *p <* 0.0001) and the number of metastatic sites (< 3, HR = 0.602, 95% Cl: 0.375–0.966, *p =* 0.035) were independently associated with longer survival outcomes (Table 4).

A total of 88 deaths occurred during the follow-up period, with the OS rate being 67.3%. The median OS for patients with low PIV was 29 months compared with 21 months in patients with high PIV (95% CI: 19.4–22.5, *p <* 0.0001; Fig. 1B).

### PIV dynamics

**Fig. 2:**
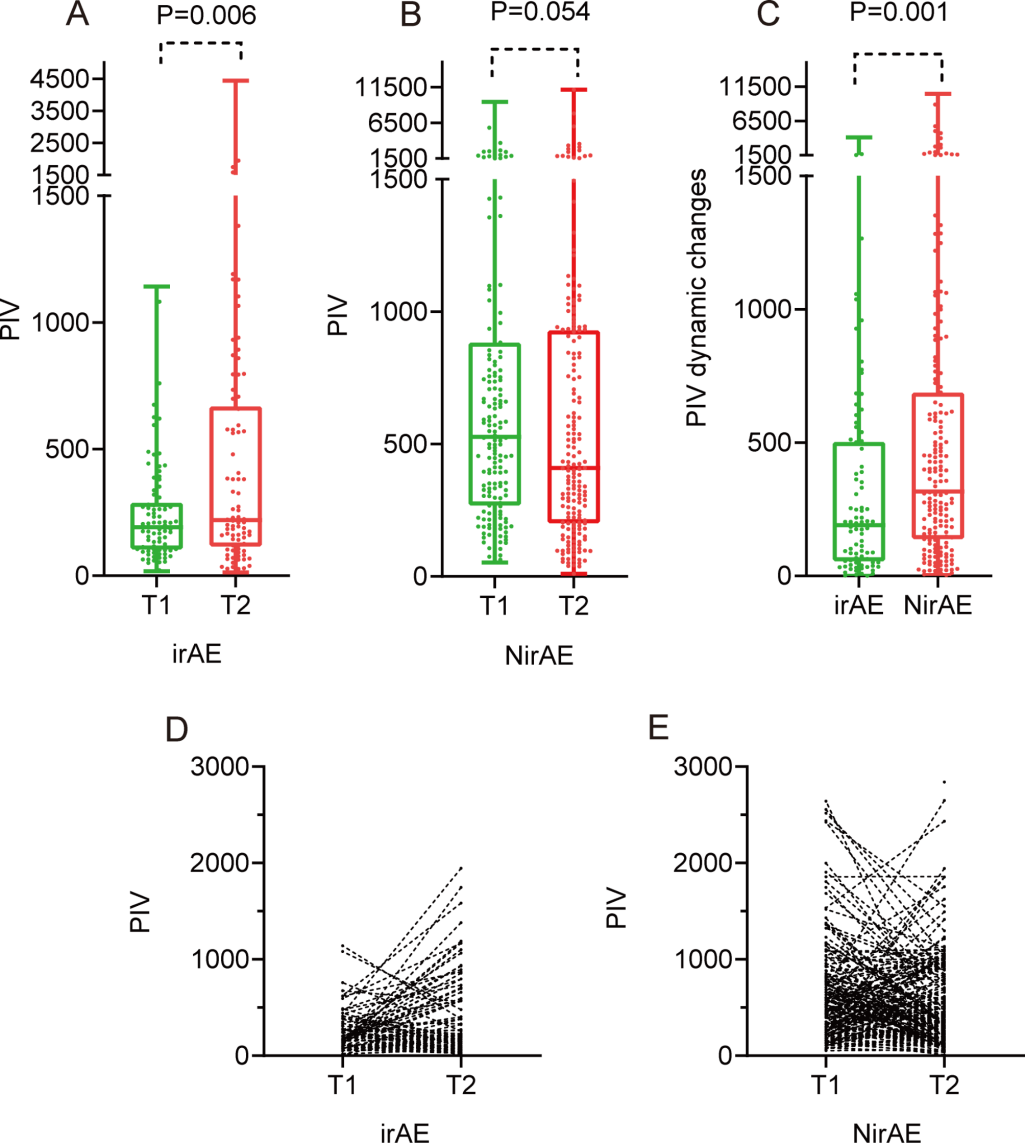
PIV dynamic changes during the immunotherapy.(**A**) Comparison of PIV at baseline and week 3–4 from irAEs patients. (**B**) Comparison of PIV at baseline and week 3–4 from no irAEs patients. (**C**) Comparison of PIV dynamics between the irAEs and no irAEs patients. (**D**) PIV dynamics during baseline and week 3–4 in irAEs patients. (1 data point is outside the axis limits) (**E**) PIV dynamics during baseline and week 3–4 in no irAEs patients. (13 data points are outside the axis limits)

A box plot plotted for comparing irAEs patients with PIV at T1 and T2 revealed a mean of 191.6 vs. 219.4 and an interquartile range (IQR) of 176.9 vs. 539.7. Moreover, the box plot suggested a significant difference in the association of PIV with irAEs between T1 and T2 (Fig. 2A, p *=* 0.006). For patients with no irAEs, the median of the box plot at T1 and T2 was 527.5 and 409.5, with the IQR being 620.8 and 727.9, respectively; however, these values did not significantly differ between the two time points (*p =* 0.056; Fig. 2B). In addition, PIV dynamics were compared between patients with irAEs and without irAEs using box plot analysis. The median obtained for patients with irAEs and without irAEs was 190.0 and 317.0, with an IQR of 447.6 and 550.3, respectively. This indicated a significant difference in PIV dynamics between the two groups (*p =* 0.001; Fig. 2C). The line chart (Fig. 2D) shows the changes in PIV with follow-up. The average change in PIV in patients with irAEs was 350 (Fig. 2D) and in those without irAEs was 678 (Fig. 2E). PIV dynamics were significantly lower in patients with irAEs than in those without irAEs.

The median PFS was longer for patients with a smaller PIV change (9 months; 95% CI: 7.9–10.0) than for patients with a greater PIV change (6 months; 95% CI: 4.8–7.1; log-rank test *p =* 0.0119; Fig. 1C). Likewise, median OS was significantly longer for patients with a smaller PIV change (28 months; 95% CI: 24.3–31.8) than for patients with a greater PIV change (21.6 months; 95% CI: 18.6–23.3; log-rank test *p =* 0.0021; Fig. 1D).

## Discussion

As a newly emerging biomarker, PIV integrates neutrophil, platelet, monocyte, and lymphocyte counts, thus reflecting the systemic and intratumoral inflammatory/ immune system status. The present retrospective study suggested that among the assessed hematological markers, only low PIV was an independent and significant factor affecting the occurrence of irAEs. Moreover, low PIV at baseline was the only independent prognostic factor of both PFS and OS, and the change in PIV dynamic before and after treatment was directly associated with the occurrence of irAEs and with clinical prognosis.

The underlying mechanisms and rationale of each peripheral blood marker are different. NLR is composed of neutrophil-to-lymphocyte ratio, PLR is composed of platelet-to-lymphocyte ratio, MLR is composed of monocyte-to-lymphocyte ratio, and SII is a comprehensive marker calculated from the three indicators of neutrophils, lymphocytes, and platelets. However, the dNLR is composed of leukocytes and neutrophilsIn. In addition, platelets play an important role in hemostasis and thrombosis; however, tumor cells may bind to platelets to escape from the immune system [32, 33]. Moreover, activated platelets release a variety of factors that promote tumor development and invasion [34]. Similar to platelets, monocytes are closely related to the occurrence and development of cancer. Studies have shown that peripheral blood monocytes can indirectly interact with tumor-associated macrophages in the TME [35], and M2 macrophages can promote the growth of tumor cells [36]. Similarly, neutrophils play a role in tumor progression by releasing reactive oxygen species and secreting pro-tumor cytokines, which induce angiogenesis, invasion, and immunosuppression [37, 38]. In contrast, lymphocytes suppress tumorigenesis, and CD8 + and CD4 + T cells in the TME mediate antitumor effects [39]. Neutrophils, platelets, and monocytes all show cancer-related inflammatory responses of patients, while lymphocytes represent the immunomodulatory status of patients in cancer treatment. Overall, in addition to dNLR, which is the main specific parameter of inflammatory response [40], the other four types of peripheral blood biomarkers are indicators of the balance between immunity and inflammation, and have certain clinical value in immunotherapy of patients with NSCLC.

The difference between the four types of indicators mentioned above is that PIV integrates neutrophils, monocytes, platelets and lymphocytes, covering as many peripheral blood parameters as possible. In theory, PIV is a more objective indicator of the complex immune and inflammatory status of the body compared with individual systemic inflammatory indicators such as SII and PLR. As PIV represents all these parameters, it is an external manifestation of a state that reflects the balance between pro-tumor and anti-tumor factors in the TME.

Notably, a previous study has shown that PIV is associated with irAEs in patients with gastrointestinal tumors; however, this role of PIV has not been discussed in patients with NSCLC. In line with this, the present study found that many blood biomarkers were correlated with the occurrence of irAEs, with PIV exhibiting a significant correlation. Unfortunately, other clinicopathological characteristics of patients, such as age, sex, and pathological subtype, were not correlated with the incidence of irAEs. Moreover, a low PIV indicated a greater anti-tumor activity. This implies that immune supplementation using ICIs would ultimately lead to increased incidence of irAEs.

The use of PIV as a predictor of cancer prognosis has been previously investigated and confirmed in a few studies [41, 42]. The present retrospective analysis supports the value of PIV in survival analysis. In addition, the Cox multifactorial analysis further confirmed that PIV is the only independent factor affecting PFS and OS. This is because PIV may capture the complexity of the immune environment and its many components more comprehensively than individual blood cell parameters or other combined statistics. Thus, a low PIV at baseline implies stronger immunity prior to treatment, which reflects the suppression of tumor growth and invasion, ultimately prolonging survival.

In fact, there have been many studies demonstrating the prognostic value of blood biomarker kinetic studies in immunotherapy [43, 44]. When patients with non-small cell lung cancer receive immunotherapy for the first time, the immune and inflammatory reactions of the body are the most intense. And the occurrence of irAEs often occurs in the process of early immunotherapy. Therefore, evaluating the immune and inflammatory response in vivo by analyzing the changes in peripheral blood parameters of patients before and after the first immunotherapy is an important indicator to determine whether patients are suitable for immunosuppressants. However, the focus of this study is not limited to pre-treatment baseline PIV. It is necessary and valuable to assess the dynamic changes in PIV, owing to its correlation with irAEs and clinical prognosis in patients with NSCLC. Cancer is a progressive disease, and baseline PIV can only describe the inflammatory/immune status of a patient over a particular time point. Our results further demonstrate that a smaller PIV change is significantly associated with the incidence of irAEs, and these patients with smaller PIV change have longer PIV and OS. Therefore, a kinetic study of PIV may reflect the variation in the inflammatory/immune system status during the short-term treatment, which theoretically provides a better picture of disease progression and treatment status. Hence, PIV kinetic studies can be used for predicting clinical survival, real-time monitoring of immunotherapy efficacy, and dynamic monitoring of TME homeostasis.

However, our study has a few limitations. This was a retrospective study and lacked prospective validation. In addition, the follow-up period was short (median follow-up: 17 months). And many clinical indicators will still affect the results, such as concomitant diseases, complications, and even the process of processing clinical specimens may affect the serum concentration of each indicator. Therefore, in this study, we tried to strictly standardize the inclusion of the population and the course of treatment as much as possible, and exclude the relevant bias as much as possible. At the same time, from a methodological point of view, we analyzed scientifically and comprehensively, and tried not to lose any valuable indicators. Even a single baseline indicator may have errors, we tried to analyze PIV from the perspective of dynamics, intercepting the test indicators at two time points before and after treatment, analyzing the changes in immunological and inflammatory values before and after treatment, so as to assess the efficacy, and exclude the possibility of data error or bias as much as possible.

This study successfully validated the role of PIV in predicting irAEs and determining clinical prognosis in patients with NSCLC receiving immunotherapy. Finally, the PIV dynamic evaluation time needs to be extended, and PIV needs to be assessed at multiple time points. This will better reflect the balance between the pro-tumor and anti-tumor factors in TME. In conclusion, the use of PIV as a blood biomarker for clinical regression and prognostic assessment in immunotherapy-treated patients with NSCLC should be further explored in prospective clinical trials with larger sample sizes and longer follow-up periods. In fact, there have been many studies combining blood biomarkers as clinical factors with other markers, such as radiomics, proteogenomics, etc. [45], and even creating reliable nomograms for the prediction of clinical immunotherapy and risk stratification [46]. Therefore, from our point of view as clinicians, our future work is to try to incorporate more clinical data, conduct multi-omics joint studies, and establish reliable predictive models for clinical decision-making with the help of PIV and PIV kinetics.

## Conclusion

In patients with NSCLC, PIV at the baseline and its dynamics can be used as early, surrogate markers to monitor immunotherapy efficacy, determine irAEs, and predict survival. The clinical application of dynamic changes in immunoinflammatory markers merits further investigation.

## Data Availability

The datasets generated and/or analyzed during the current study are available from the corresponding author on reasonable request.
